# A novel robust meta-analysis model using the *t* distribution for outlier accommodation and detection

**DOI:** 10.1017/rsm.2025.8

**Published:** 2025-03-13

**Authors:** Yue Wang, Jianhua Zhao, Fen Jiang, Lei Shi, Jianxin Pan

**Affiliations:** 1 School of Statistics and Mathematics, Yunnan University of Finance and Economics, Kunming, China; 2 Guangdong Provincial Key Laboratory of Interdisciplinary Research and Application for Data Science, BNU-HKBU United International College, Zhuhai, China

**Keywords:** expectation maximization, meta-analysis, outlier accommodation, outlier detection, robustness

## Abstract

Random effects meta-analysis model is an important tool for integrating results from multiple independent studies. However, the standard model is based on the assumption of normal distributions for both random effects and within-study errors, making it susceptible to outlying studies. Although robust modeling using the *t* distribution is an appealing idea, the existing work, that explores the use of the *t* distribution only for random effects, involves complicated numerical integration and numerical optimization. In this article, a novel robust meta-analysis model using the *t* distribution is proposed (*t*Meta). The novelty is that the marginal distribution of the effect size in *t*Meta follows the *t* distribution, enabling that *t*Meta can simultaneously accommodate and detect outlying studies in a simple and adaptive manner. A simple and fast EM-type algorithm is developed for maximum likelihood estimation. Due to the mathematical tractability of the *t* distribution, *t*Meta frees from numerical integration and allows for efficient optimization. Experiments on real data demonstrate that *t*Meta is compared favorably with related competitors in situations involving mild outliers. Moreover, in the presence of gross outliers, while related competitors may fail, *t*Meta continues to perform consistently and robustly.

## Highlights

### What is already know

Random effects model is a popular tool for handling heterogeneity between studies in meta-analysis. However, the standard model is based on the Gaussian assumption and thus is susceptible to outlying studies.

### What is new

A novel robust meta-analysis model using student’s *t* distribution called *t*Meta is proposed, which is capable of simultaneously accommodating and detecting outlying studies in a simple and adaptive manner. Empirical results show that *t*Meta is compared favorably with related competitors.

### Potential impact for *Research Synthesis Methods* readers

Compared with related competitors, *t*Meta frees from numerical integration and allows for efficient optimization, which, to our knowledge, offers the first neat solution to robust meta-analysis modeling using the *t* distribution. Importantly, *t*Meta provides a simple but powerful robust meta-analysis tool that can accommodate and detect both mild and gross outliers simultaneously.

## 1 Introduction

In meta-analyses, the collected studies often exhibit heterogeneity, characterized by greater variation among studies than can be explained by the variation within each study,^1^ which could result in misleading conclusions about the overall treatment effect.^2^^,^ ^3^ The random effects model is a popular tool for handling heterogeneity.^4^^,^ ^5^ However, the standard model assumes normal distributions for both random effects and within-study errors (nMeta), making it susceptible to outlying studies.

Outlier detection is a central research area in meta-analysis. Many methods have been developed. For example, a likelihood ratio test was constructed to identify outliers by detecting inflated variance^6^; a forward search algorithm was developed specifically for this purpose^7^; several outlier and influence diagnostic procedures in meta-regression models were presented.^8^ Subsequently, case deletion diagnostics and local influence analysis using multiple perturbation schemes, were investigated.^9^ Several Bayesian outlier detection measures were also introduced for handling outlying studies in network meta-analysis.^10^ Another important methodology for dealing with outliers is outlier accommodation or robust estimation, which can down-weight the influence of outliers. For instance, robust functions like Huber’s rho and Tukey’s biweight functions were employed to replace the original non-robust objective function, resulting in robust estimates.^11^

This article focuses on outlier accommodation and detection simultaneously. Several efforts have been made toward this objective. Non-normal alternatives to normal random effects were investigated, and it was found that the *t* distribution performs the best (*t*RE-Meta).^12^ The shortcoming is that the marginal distribution of 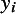 in *t*RE-Meta is mathematically intractable. Consequently, numerical integration is required to evaluate the log-likelihood and numerical optimization methods have to be employed for maximum likelihood (ML) estimation. Subsequently, new models where 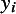 has a tractable marginal distribution were presented, including the three parameter symmetric marginal model (SYM-Meta) and the four parameter skew marginal model (SKM-Meta).^13^ Nevertheless, numerical optimization has still to be employed to obtain ML estimates. As a tractable model, a variant of a two-component mixture model (MIX-Meta) was proposed, with one component modeling standard studies and the other addressing outlying studies.^1^ In MIX-Meta, the marginal distribution of the observed effect 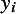 is a mixture of two normal distributions. However, MIX-Meta suffers from initialization issues, necessitating multiple runs of the fitting algorithm with different starting values.

The common feature of these methods is that the error terms are assumed to follow the normal distribution. In this article, we break this limitation as the marginal distribution of error term in our proposed model follows the *t* distribution. It is known that the *t* distribution includes the normal distribution as a special case when the degrees of freedom 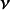 goes to infinity. This means that *t*Meta offers greater flexibility and applicability than the conventional normal assumption. The main contributions of this article are as follows.

- The marginal distribution of the effect size 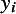 in *t*Meta follows the *t* distribution, enabling it to simultaneously accommodate and detect outliers in a simple and adaptive manner. 1) The *t* distribution offers an additional robustness tuning parameter which can adaptively down-weight outlying studies. 2) The expected weights follow in proportion to a Beta distribution, providing a useful critical value for outlier detection.
- *t*Meta provides a simple but powerful robust meta-analysis tool that can accommodate and detect both mild and gross outliers simultaneously. As can be seen from Section 4, 1) *t*Meta versus SYM-Meta and SKM-Meta. Both the three-parameter SYM-Meta and four-parameter SKM-Meta fail in most of the outlier detection tasks, though they have good performance in outlier accommodation. 2) *t*Meta versus *t*RE-Meta and MIX-Meta. While all the three methods can be used to detect mild outliers, *t*Meta performs the best in outlier accommodation. More importantly, in the presence of gross outliers, both *t*RE-Meta and MIX-Meta could fail while *t*Meta still performs satisfactorily.
- Due to its mathematical tractability, *t*Meta frees from numerical integration and allows for efficient optimization. In contrast, *t*RE-Meta requires both complicated numerical integration and numerical optimization; SYM-Meta and SKM-Meta involve complex numerical optimization^13^; MIX-Meta requires multiple runs of the fitting algorithm due to the sensitivity issue of mixture models to initialization.^1^ To our knowledge, *t*Meta offers the first neat solution to robust meta-analysis modeling using the *t* distribution.

The rest of this article is organized as follows. Section 2 reviews some related works. Section 3 proposes our new model *t*Meta. Section 4 conducts case studies to compare *t*Meta with several closely related competitors. Section 5 offers a summary of the entire article.

## 2 Background

In this section, we briefly review some fundamental results concerning the standard model nMeta and Student’s *t* distribution.

### 2.1 Normal meta-analysis model (nMeta)

In nMeta, the effect size 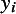 for the *i*-th study is defined as follows (1)

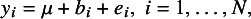

where the random effects 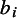 captures heterogeneity across studies and follows 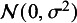 , the within-study error 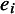 follows 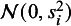 and they are independent of each other. Here, 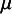 is the overall effect size, 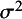 is the unknown between-study variance and 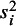 is the known within-study variance.

From (1), we have 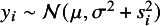 . Estimates for the parameters 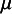 and 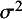 can be obtained through maximum likelihood methods.^14^

### 2.2 Student’s *t* distribution

Suppose that a random variable *y* follows the univariate *t* distribution 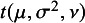 , with center 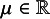 , scale parameter 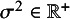 , and degrees of freedom 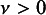 , then the probability density function (p.d.f.) of *y* is given by 

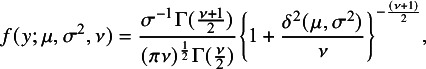

where 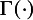 is the gamma function and 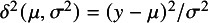 is the squared Mahalanobis distance of *y* from the center 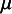 with respect to 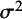 . If 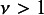 , 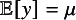 ; if 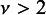 , Var 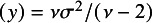 ; and if 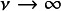 , 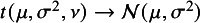 .^15^

Given a latent weight variable 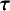 distributed as the Gamma distribution 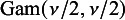 , *y* can also be represented hierarchically as a latent variable model as follows^15^: (2)

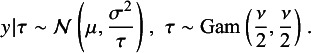



Under model (2), it is easy to obtain the marginal distribution 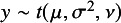 by 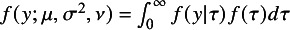 ^16^ and the posterior distribution of 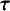 given *y*


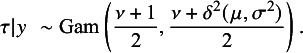



## 3 Novel robust meta-analysis model

In this section, we propose a novel robust meta-analysis model called *t*Meta. In Section 3.1, we present the model. In Section 3.2, we develop an algorithm for parameter estimation. In Section 3.3 and Section 3.4, we give the details for outlier accommodation and detection in *t*Meta.

### 3.1 The proposed tMeta model

Based on the hierarchical representation of the *t* distribution in Section 2.2, we propose a novel robust random effects meta-analysis model, denoted by *t*Meta. Its latent variable model can be expressed by (3)

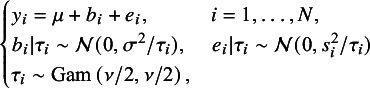

where, unlike nMeta, the random effects 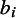 and the within-study error 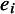 under *t*Meta are only conditionally independent; that is, 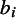 and 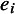 are mutually independent given the latent weight 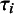 ; 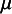 is the overall effect size, 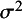 is the unknown between-study variance, 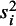 is the known within-study variance, and the degrees of freedom 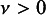 .

According to (3), integrating out the latent weight 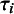 yields the marginal distributions 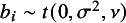 and 
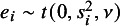
 . Furthermore, using the property of the normal distribution, it is easy to obtain the conditional distribution of 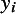 given 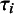


(4)

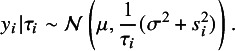

Integrating out the latent weight 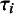 , we obtain an important result that the marginal distribution 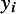 follows a *t* distribution, that is, (5)

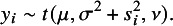



Note that this result is not available under *t*RE-Meta model, where the marginal distribution of 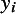 is mathematically intractable. This difference arises because *t*Meta and *t*RE-Meta model outliers in distinct ways. In *t*RE-Meta, outliers are assumed to result solely from extreme variation within studies. By contrast, as shown in (4), *t*Meta models the importance of a study *i* at the 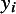 -level by incorporating a latent weight 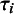 associated with 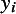 to reflect the study’s significance. The same 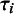 is then naturally applied to both the between-study effect 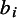 and the within-study error 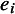 , as shown in (3). In other words, outliers in *t*Meta are assumed to result from extreme variation across both the within-study and between-study levels. This hierarchical modeling framework enables a tractable marginal model for the effect 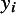 .

As a result, the degrees of freedom 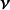 in *t*Meta can be interpreted as an overall measure of deviation from the nMeta model across both within-study and between-study levels. The two models differ significantly when 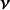 is small but become similar as 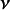 becomes large. Similar overall measures have appeared in the literature; for example, a total correlation parameter has been used to capture overall correlation across both levels in the normal random-effects model.^17^ Notably, nMeta emerges as a special case of *t*Meta in the limit, as the *t* distribution 
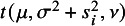
 approaches the normal distribution 
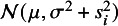
 as 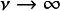 .

#### 3.1.1 Probability distributions

From *t*Meta model (3), it is easy to obtain the following probability distributions (6)

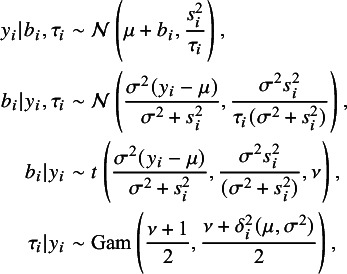

where (7)

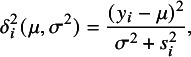

is the squared Mahalanobis distance of 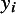 from the overall effect size 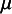 . It is clear that all the probability distributions under *t*Meta, including the marginal distributions of 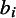 , 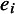 and 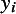 given in Section 3.1, are well-known and tractable.

#### 3.1.2 Robust meta-regression with covariates

When several covariates are involved, the model (3) can be extended to a more general model, 

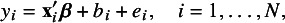

where 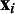 represents *p*-dimensional vector of covariates, 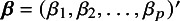 is the *p*-dimensional regression coefficients; the random variables 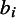 and 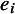 and the other parameters 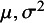 and 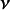 are similar as those in *t*Meta (3). Under this model, we have 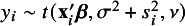 .

### 3.2 Maximum likelihood estimation

In this section, we develop estimation algorithms for obtaining the ML estimates of the parameters 
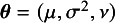
 in the *t*Meta model. Given the effect size vector 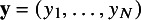 , from (3) the observed data log-likelihood function 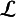 is (up to a constant), (8)

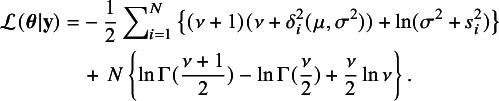



The maximization of 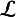 in (8) can be obtained by standard numerical optimizers. However, we shall propose an EM-type algorithm to obtain the ML estimate 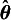 because of its simplicity and stability.^15^ From (6), the required conditional expectation in the E-step can be obtained as (9)

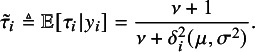

The details about the development of this algorithm can be found in Section A.1 of the Appendix.

### 3.3 Outlier accommodation

#### 3.3.1 Adaptive outlier accommodation

Looking at (9), (A.2), and (A.3), the following can be observed.

- When the data contain no outliers and the 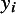 ’s come from nMeta, 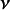 is expected to take on large values. This causes all the weights 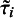 in (9) to be close to 1. Consequently, (A.2) and (A.3) would degenerate to those of nMeta, and hence *t*Meta adaptively degenerates to nMeta in this case.
- In the presence of outliers, 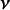 is expected to take on small values, and the outlying study 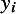 would have a much greater squared Mahalanobis distance 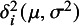 compared with non-outliers, causing the outlier’s 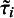 in (9) to be much smaller than those of non-outliers. Consequently, the impact of outliers on the estimators in (A.2) and (A.3) is substantially reduced, allowing *t*Meta to yield robust estimates.

In summary, the degrees of freedom 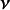 is a robustness tuning parameter that adapts according to the presence of outliers in the data.

#### 3.3.2 Breakdown point

In statistics, the robustness of estimators is assessed by breakdown points, which are the proportion of arbitrarily large outlying observations an estimator can tolerate before giving an incorrect result. The following Proposition 1 gives the breakdown point of *t*Meta.

Proposition 1. The upper bound of the breakdown point of *t*Meta is 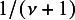 .

Proof. As proved by Dümbgen and Tyler,^18^ the upper bound of the breakdown point of the *d*-dimensional multivariate *t* distribution is 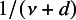 . For *t*Meta, the dimension of *t*-distributed 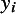 is 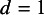 and hence the upper bound of *t*Meta is given by 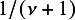 . This completes the proof.

In our implementation, we restrict 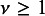 . Proposition 1 shows that *t*Meta is a highly robust method as its breakdown point could be close to 50% under this restriction.

### 3.4 Outlier detection

Similar to that in multivariate *t* and matrix-variate *t* distributions,^19^^–^ ^21^ the expected weight 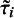 in *t*Meta given by (9) can be used as outlier indicator. Let (10)

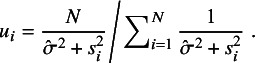

The following Proposition 2 gives the details.

Proposition 2. Assume that the study 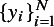 follow *t*Meta model (3). Given the ML estimate 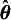 , we have, when the estimate 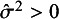 , 

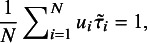

and when 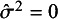 , 

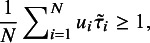

Proof. The proof can be found in Section A.2 of the Appendix.

Proposition 2 shows that when the estimate 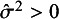 , the average of all 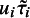 ’s equals to 1. In other words, the study with 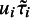 much smaller than 1 (i.e., 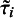 much smaller than 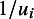 ) or close to 0 can be considered as an outlier. When 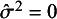 , our experience reveals that 
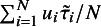
 may be slightly greater than 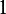 .

In practice, a critical value is needed to judge whether a study is an outlier or not. The following Proposition 3 does this task. Let 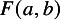 and 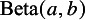 stand for the *F* distribution and Beta distribution with parameters *a* and *b*, respectively. The 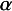 quantile of 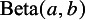 is denoted by 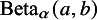 .

Proposition 3. Suppose that the study set 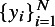 follow *t*Meta model (3). Then we have that the Mahalanobis distance 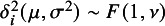 . Given the ML estimate 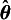 , the weights 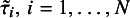 converge in distribution to 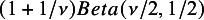 as the study sample size *N* approaches infinity. Therefore, at a significance level of 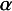 , the *i*-th study with 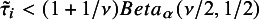 could be identified as an outlier.

Proof. This is a special case with dimension 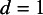 of the result on the *d*-dimensional multivariate *t* distribution proved by Wang and Fun.^19^ This completes the proof.

## 4 Results

In this section, we compare the performance of our proposed *t*Meta with five closely related methods: nMeta, *t*RE-Meta, MIX-Meta, SYM-Meta and SKM-Meta using four benchmark real-world datasets. For *t*Meta, the iteration stops when the relative change in the objective function 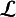 (|1- 
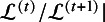
 ) is smaller than the given threshold 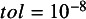 or the number of iterations exceeds 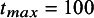 . For nMeta, *t*RE-Meta, and MIX-Meta, we use the R codes available from https://cran.r-project.org/web/packages/metaplus/. In addition, we use the default setting for MIX-Meta, i.e., 20 initializations. The code for SYM-Meta and SKM-Meta can be found from the supplementary materials by Baker and Jackson.^13^

To perform outlier detection for *t*Meta, we utilize the critical value provided in Proposition 3 and set the significance level 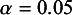 . For better visualization, we equivalently plot the inverse of 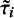 . That is, the study with 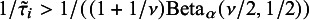 is identified as an outlier for *t*Meta. For MIX-Meta, we use the empirical threshold 0.9 as suggested by Beath,^1^ which represents the posterior probability that a study belongs to the outlying component. For SYM-Meta and SKM-Meta, we adopt the *p*-value method specially developed for both models by Baker and Jackson.^13^ Since *t*RE-Meta lacks guidelines for setting the threshold, we follow the empirical approach by Baker and Jackson,^12^ treating studies with very small values of the relative weight 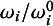 , or equivalently, very large values of 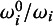 as outliers, where 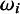 and 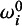 are the weights under *t*RE-Meta and nMeta, respectively.

To compare the computational efficiency, we will report their total CPU time consumed by various methods, which is sum of the time used for training model and that for detecting outliers. For *t*Meta and MIX-Meta, outlier detection is a byproduct of the model training and incurs no additional time cost. However, *t*RE-Meta, SYM-Meta and SKM-Meta require additional time cost for outlier detection. To be specific, *t*RE-Meta requires numerical methods to compute 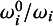 while SYM-Meta and SKM-Meta necessitate additional efforts to implement the *p*-value method.

### 4.1 Intravenous magnesium

**Figure 1:**
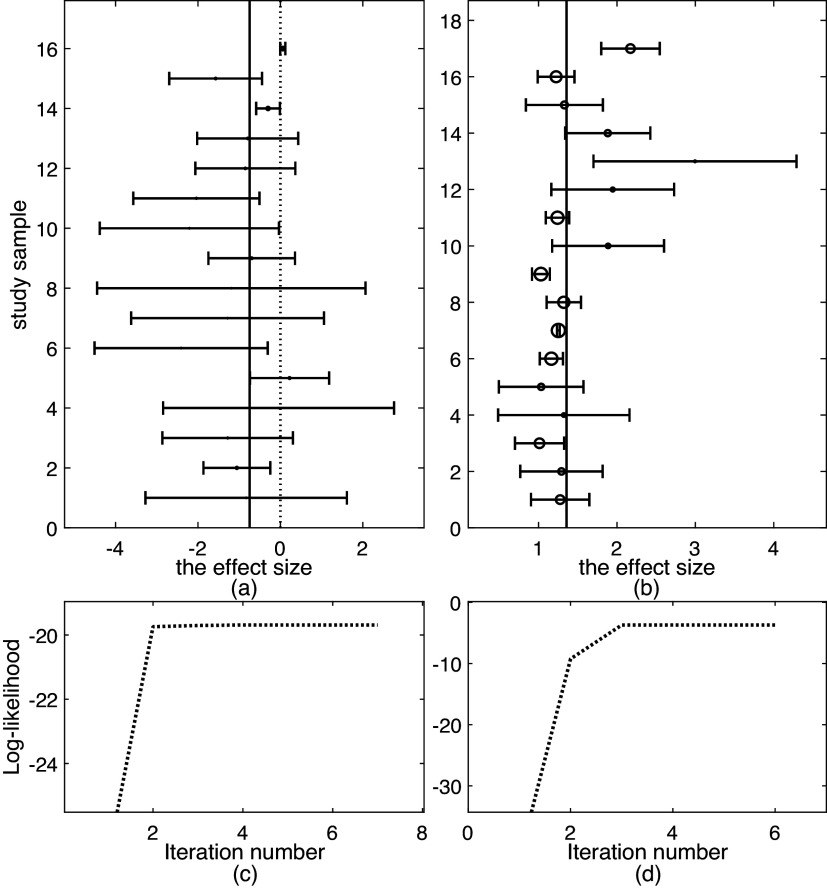
*Top row: forest plots on two datasets: (a) Mag and (b) Hipfrac, where each effect size* 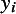 *and 95% confidence interval are shown as circle and solid line, respectively. Bottom row: evolement of log-likelihood of* 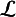 *versus number of iterations: (c) Mag and (d) Hipfrac*.

The Mag dataset^22^ comprises 16 studies. As can be seen from the forest plot shown in Figure 1(a), it looks difficult to visually identify which study is an outlier except that study 16 seems different from others due to its relatively large 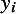 value and low 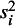 . Previous researches^1^^,^ ^6^ have analyzed this dataset and found no outliers. Below we perform outlier detection with various methods.

We fit all the six methods on the Mag dataset. Table 1 collects the results. The results in Table 1 show all the six methods yield similar performance. This means that all the five methods *t*RE-Meta, MIX-Meta, SYM-Meta, SKM-Meta and *t*Meta could degrade to nMeta. Nevertheless, among the five robust methods, *t*Meta is computationally the most efficient while *t*RE-Meta and MIX-Meta require much more time. Figure 1(c) shows the evolvement of log-likelihood 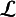 versus number of iterations when fitting *t*Meta. It can be seen from Figure 1(c) that *t*Meta converges within 7 iterations on this dataset.

**Table 1:** Results of parameter estimates, negative log-likelihood, and CPU time (in seconds) by various methods on Mag dataset

Methods	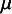	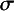	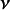	- 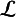	Time
nMeta	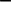 0.746	0.504	—	19.685	—
*t*RE-Meta	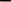 0.746	0.504	inf	19.685	3.0
MIX-Meta	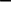 0.746	0.504	—	19.685	32.4
SYM-Meta	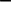 0.746	0.504	—	19.685	0.3
SKM-Meta	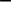 0.746	0.504	—	19.685	0.3
*t*Meta	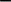 0.746	0.504	inf	19.685	0.05

*Note*: ‘—’ indicates that a method does not have corresponding results.

**Figure 2:**
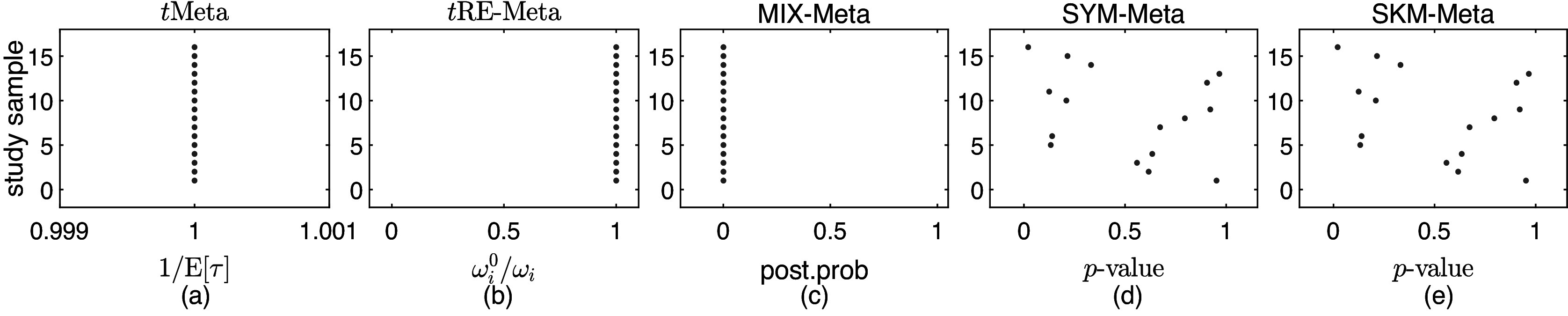
*Results on outlier detection by various methods on Mag dataset: (a) *t*Meta; (b) *t*RE-Meta; (c) MIX-Meta; (d) SYM-Meta; (e) SKM-Meta. The marker solid point* 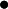 *in blue represents normal studies judged by a method*.

Figure 2 shows the results of detecting outliers by the five methods. It can be seen from Figure 2 that all the five methods suggest no outliers for Mag dataset. This finding is consistent with that by Beath.^1^

### 4.2 Hipfrac dataset

The Hipfrac dataset^23^ contains 17 studies, collected from an investigation on the magnitude and duration of excess mortality after hip fracture among older men. Figure 1(b) shows the forest plot, from which it seems hard to identify which study is an outlier. Below we perform outlier analysis with various methods.

We fit all the six methods on the Hipfrac dataset. Table 2 summarizes the results. The results in Table 2 show that *t*Meta and SKM-Meta obtain significantly better BIC than the other methods and SKM-Meta wins by a narrow margin. In terms of computational efficiency among the five robust methods, *t*Meta is the fastest while *t*RE-Meta and MIX-Meta are the slowest runners. Figure 1(d) shows the evolution of log-likelihood 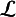 versus number of iterations when fitting *t*Meta. It can be seen from Figure 1(d) that *t*Meta converges within 6 iterations on this dataset.

**Table 2:** Results of parameter estimates, negative log-likelihood, BIC, and CPU time (in seconds) by various methods on Hipfrac dataset

Methods	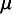	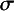	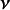	- 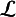	BIC	Time
nMeta	1.357	0.260	—	8.498	22.661	—
*t*RE-Meta	1.251	0.013	0.582	6.575	21.649	79.2
MIX-Meta	1.252	0.000	—	4.507	20.347	364.1
SYM-Meta	1.220	0.074	—	5.670	19.840	0.222
SKM-Meta	1.202	0.063	—	1.439	**14.212**	0.2
*t*Meta	1.252	0.000	1.871	3.700	15.899	0.03

*Note*: The best method is shown in boldface. ‘—’ indicates that a method does not have corresponding results.

**Figure 3:**
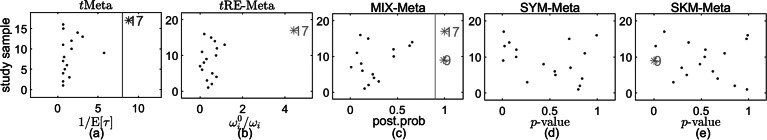
*Results on outlier detection by various methods on Hipfrac dataset: (a) *t*Meta; (b) *t*RE-Meta; (c) MIX-Meta; (d) SYM-Meta; (e) SKM-Meta. The vertical line indicates the critical value for *t*Meta (red) and the threshold 0.9 (magenta) for MIX-Meta. The vertical line indicates the critical value for *t*Meta and the threshold 0.9 for MIX-Meta. The marker solid point* 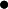 in blue represents normal studies judged by a method. Star ‘*’ signals outlying studies, with red for *t*Meta and magenta for the other methods.

Figure 3 shows the results of detecting outliers by the five methods. It can be seen from Figure 3 that both *t*Meta and *t*RE-Meta identify study 17 as an outlier. This result is consistent with that obtained by Lin et al.^2^ In contrast, MIX-Meta identifies one more outlier: study 9, while SYM-Meta and SKM-Meta fail completely.

It is interesting to make a further comparison between *t*Meta and *t*RE-Meta. From Figure 3(a), it can be seen that *t*Meta detects that study 9 as being close to an outlier, while it is not the case by *t*RE-Meta from Figure 3(b). In fact, Lin et al.^2^ have considered study 9 as a potential outlier and perform a sensitivity analysis by removing this study. As a result, they found that study 9 is not influential. Therefore, the result by *t*Meta is well match that obtained by Lin et al.^2^

### 4.3 Fluoride toothpaste

This dataset contains 70 studies, obtained from an evaluation of fluoride’s efficacy in preventing childhood dental caries.^24^ The effect size 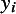 denotes the difference between control and treatment groups, with negative values signifying significant therapeutic effects.

Previous works^1^^,^ ^6^^,^ ^12^ have concluded that there exist three outliers in this dataset: study 63, study 50 and study 38. Contrarily, the analysis with SKM-Meta suggests no outliers in the dataset.^13^ To better examine the outlier detection performance by various methods, we shall perform two experiments in this section. In the first experiment of Section 4.3, we use the original dataset (Flu). In the second experiment of Section 4.3, we add the original dataset with one more artificial outlier. The resulting dataset is called modified Flu for clarity.

#### 4.3.1 Original Flu

**Figure 4:**
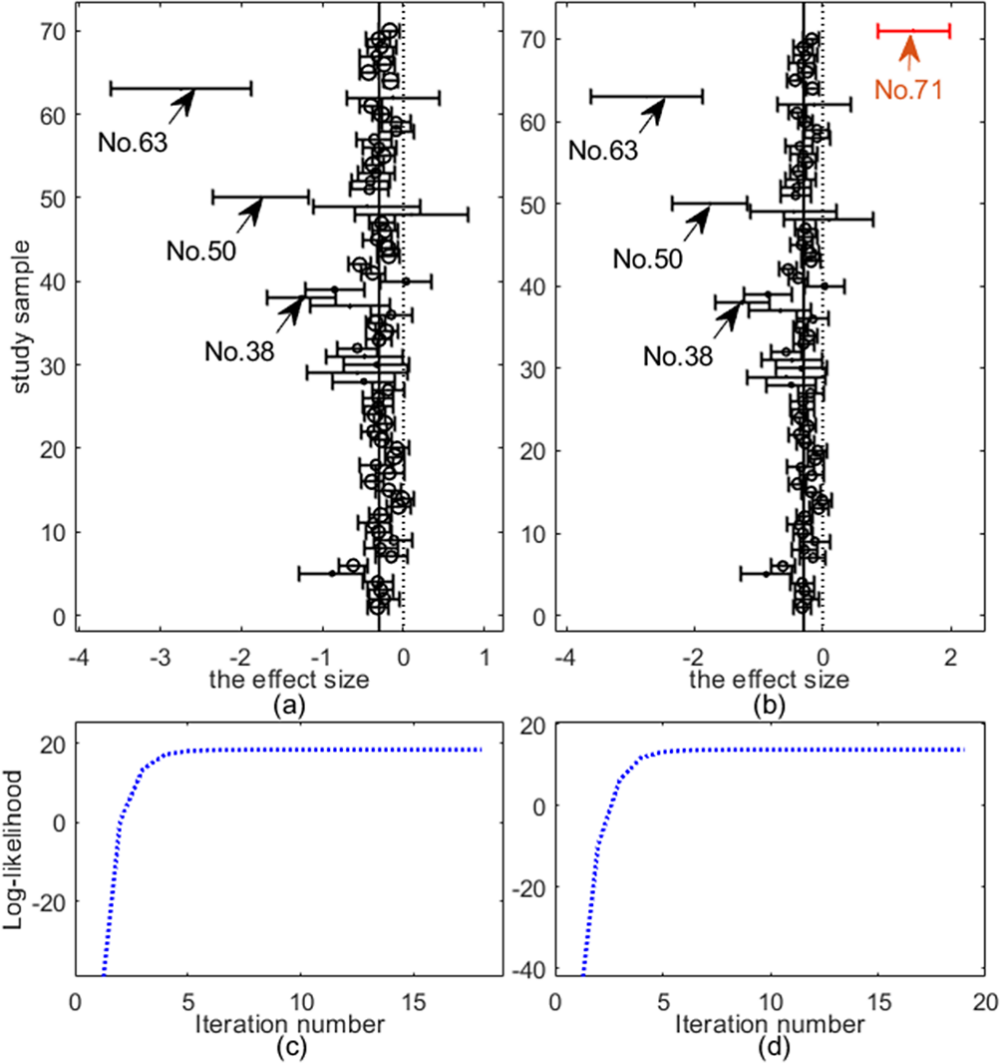
*Top row: forest plots on the fluoride toothpaste dataset: (a) Flu and (b) modified Flu, where each effect size* 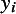 *and 95% confidence interval are shown as circle and solid line, respectively. Bottom row: evolement of log-likelihood of* 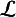 *versus number of iterations: (c) Flu and (d) modified Flu*.

Figure 4(a) shows the forest plot of the original dataset Flu. It can be observed from Figure 4(a) that studies 38, 50, and 63 look like abnormal. We then perform further analysis to identify outliers.

We fit all the six methods on Flu. Table 3 summarizes the results. The results in Table 3 show that *t*Meta, SYM-Meta and SKM-Meta obtain substantially better BIC than the other methods and SKM-Meta is again the best. Among the five robust methods, *t*Meta is the most computationally efficient while *t*RE-Meta and MIX-Meta are the most inefficient. Figure 4(c) shows the evolution of log-likelihood 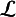 versus number of iterations when fitting *t*Meta. It can be seen from Figure 4(c) that *t*Meta converges within 18 iterations on this dataset.

**Table 3:** Results by various methods on the original and modified fluoride toothpaste dataset, including parameter estimates, negative log-likelihood, BIC, and CPU time (in seconds)

Methods	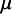	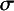	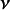	- 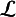	BIC	Time
	Original Flu					
nMeta	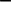 0.300	0.119	—	1.233	10.963	—
*t*RE-Meta	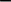 0.280	0.049	1.158	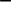 13.121	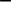 13.497	64.0
MIX-Meta	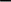 0.281	0.090	—	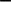 14.636	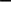 12.277	27.0
SYM-Meta	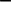 0.282	0.092	—	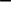 17.148	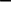 21.551	0.4
SKM-Meta	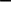 0.273	0.081	—	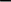 21.914	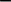 **26.834**	0.6
*t*Meta	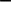 0.282	0.051	2.754	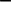 18.283	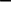 23.820	0.05
	Modified Flu					
nMeta	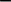 0.297	0.139	—	15.760	40.046	—
*t*RE-Meta	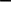 0.279	0.047	1.023	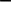 7.774	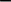 2.761	59.2
MIX-Meta	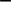 0.280	0.088	—	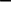 10.062	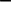 3.072	26.9
SYM-Meta	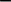 0.282	0.092	—	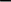 12.399	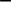 12.010	0.7
SKM-Meta	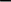 0.277	0.088	—	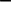 13.144	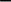 9.238	0.6
*t*Meta	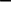 0.281	0.047	2.367	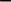 13.791	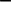 **14.794**	0.05

*Note*: The best method is shown in boldface. ‘—’ indicates that a method does not have corresponding results.

**Figure 5:**
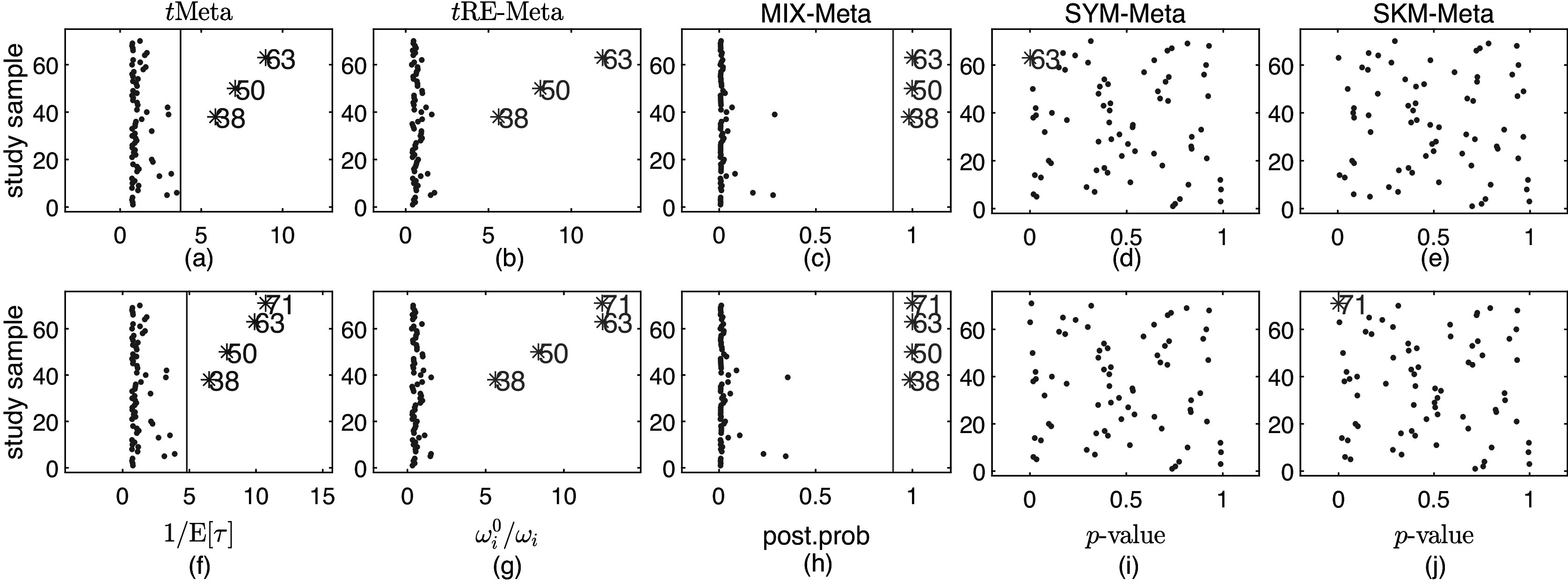
*Results on outlier detection by various methods on fluoride toothpaste dataset. Top row: the original dataset; Bottom row: the modified dataset. (a), (f) *t*Meta; (b), (g) *t*RE-Meta; (c), (h) MIX-Meta; (d), (i) SYM-Meta; (e), (j) SKM-Meta. The vertical line indicates the critical value for *t*Meta and the threshold 0.9 for MIX-Meta. The marker solid point* 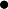 in blue represents normal studies judged by a method. Star ‘*’ signals outlying studies, with red for *t*Meta and magenta for the other methods.

The top row in Figure 5 shows the results of detecting outliers by the five methods. It can be seen from Figure 5 that *t*Meta, *t*RE-Meta and MIX-Meta all identify three studies: 63, 50, 38. This means that the result by *t*Meta is consistent with those in previous works.^1^^,^ ^6^^,^ ^12^ In contrast, SYM-Meta only detects the most abnormal study 63 as one outlier while SYM-Meta identify no outlier.

#### 4.3.2 Modified Flu

In the modified Flu, the outlier (study 71) is introduced as follows. The effect size 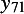 is generated from the uniform distribution *U* on the interval 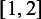 , i.e., 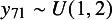 . We set its within-study variance 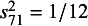 . Figure 4(b) shows the forest plot of modified Flu, from which it can be seen that the newly added study 71 looks like a mild outlier as it is very different from all the other studies.

Table 3 summarizes the results by six methods. The results in Table 3 show that *t*Meta yields the best BIC on this dataset, which is then followed by SYM-Meta, and SKM-Meta is the third best. Among the five robust methods, *t*Meta is again the best performer in computational efficiency while *t*RE-Meta and MIX-Meta are still the most inefficient. Figure 4(d) shows the evolution of log-likelihood 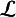 versus number of iterations when fitting *t*Meta. It can be seen from Figure 4(d) that *t*Meta converges within 19 iterations on this dataset.

The bottom row in Figure 5 shows the results of detecting outliers by the five methods. It can be seen from the bottom row of Figure 5 that *t*Meta, *t*RE-Meta and MIX-Meta successfully identify four outliers: 71, 63, 50, 38. In contrast, SYM-Meta fails to detect any outlier, while SKM-Meta can detect the newly added study 71.

### 4.4 CDP-choline

The CDP-choline dataset^25^ is obtained by exploring the cytidinediphosphocholine analysis in cognitive and behavioural disorders associated with chronic brain diseases in the elderly. The sample size is 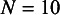 .

Previous analyses^1^^,^ ^6^^,^ ^12^ have concluded that there is one outlier in this dataset: study 8. Like Section 4.3, we perform two experiments. In the first experiment of Section 4.4, we use the original dataset (CDP). In the second experiment of Section 4.4, we modify CDP so that it contains more outliers, which is denoted by modified CDP for clarity.

#### 4.4.1 Original CDP

**Figure 6:**
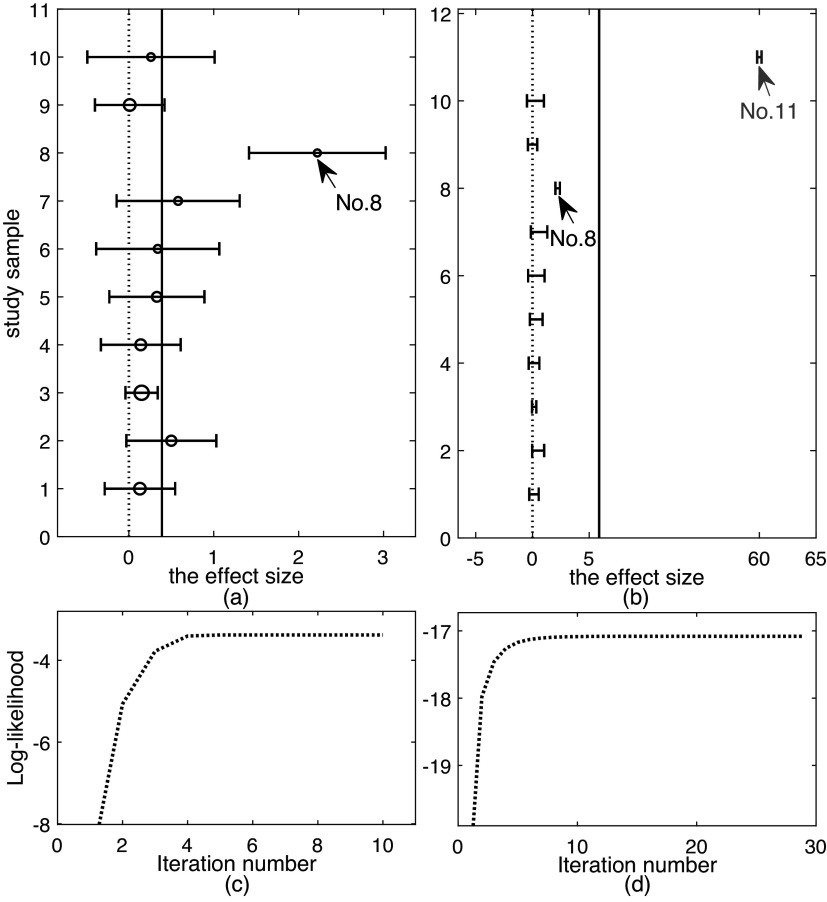
*Top row: forest plots on CDP-choline dataset: (a) original dataset; (b) modified dataset. Bottom row: evolement of log-likelihood of* 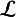 *versus number of iterations: (c) original dataset and (d) modified dataset*.

Figure 6(a) shows the forest plot of the original CDP. It can be observed from Figure 6(a) that study 8 looks like abnormal. We then perform further analysis to identify outliers.

**Figure 7:**
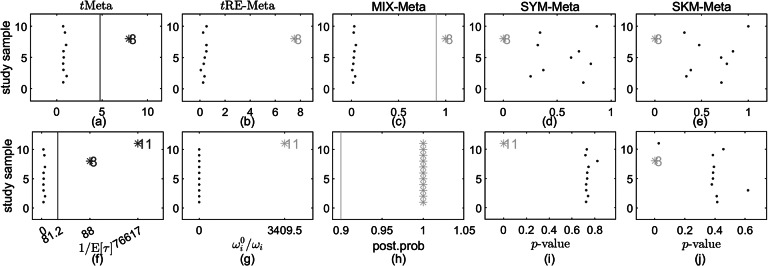
*Results on outlier detection by various methods on CDP-choline dataset. Top row: the original dataset; Bottom row: the modified dataset. (a), (f) *t*Meta; (b), (g) *t*RE-Meta; (c), (h) MIX-Meta; (d), (i) SYM-Meta; (e), (j) SKM-Meta. The vertical line indicates the critical value for *t*Meta and the threshold 0.9 for MIX-Meta. The marker solid point* 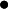 in blue represents normal studies judged by a method. Star ‘*’ signals outlying studies, with red for *t*Meta and magenta for the other methods.

We fit all the six methods on CDP. Table 4 summarizes the results. The results in Table 4 show that *t*Meta, SYM-Meta and SKM-Meta obtain significantly better BIC than the other methods and SKM-Meta is again the best. In terms of computational efficiency among the five robust methods, *t*Meta is the most efficient while *t*RE-Meta and MIX-Meta are the slowest runners. Figure 6(c) shows the evolution of log-likelihood 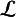 versus number of iterations when fitting *t*Meta. It can be seen from Figure 6(c) that *t*Meta converges within 10 iterations on this dataset.

**Table 4:** Results by various methods on the CDP-choline dataset, including parameter estimates, negative log-likelihood, BIC, and CPU time (in seconds)

Methods	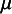	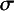	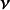	- 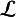	BIC	Time
	Original CDP					
nMeta	0.389	0.383	—	8.199	21.002	—
*t*RE-Meta	0.195	0.006	0.494	4.058	15.024	24.7
MIX-Meta	0.191	1.777	—	3.007	15.225	47
SYM-Meta	0.194	0.000	—	2.847	12.602	0.14
SKM-Meta	0.193	0.000	—	1.403	**12.016**	0.2
*t*Meta	0.187	0.000	2.380	3.377	13.662	0.03
	Modified CDP					
nMeta	5.879	17.126	—	46.855	98.506	—
*t*RE-Meta	0.193	0.002	0.273	13.768	**34.729**	65.9
MIX-Meta	5.879	2.455	—	46.855	103.302	19.5
SYM-Meta	5.880	17.117	—	46.855	100.904	0.3
SKM-Meta	0.484	0.711	—	21.622	52.836	0.4
*t*Meta	0.200	0.115	1.000	17.081	41.355	0.03

*Note*: The best method is shown in boldface. ‘—’ indicates that a method does not have corresponding results.

The top row in Figure 7 shows the results of detecting outliers by the five methods. It can be seen from Figure 5 that all the five methods successfully identify study 8 as an outlier. This means that the results by *t*Meta, SYM-Meta and SKM-Meta are consistent with those in previous works.^1^^,^ ^6^^,^ ^12^

#### 4.4.2 Modified CDP

In the modified CDP, we make two modifications: (i) add one outlier, namely study 11, which is set as 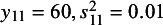 ; (ii) set 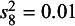 . Figure 6(b) shows the forest plot of modified CDP, from which it can be seen that the newly added study 11 is a gross outlier as it is extremely different from all the other studies and study 8 is a mild outlier but now it has a smaller within-study variance than that in the original CDP.

Table 4 summarizes the results by six methods. The results in Table 4 show that *t*RE-Meta and *t*Meta have better BIC than the other methods on this dataset and *t*RE-Meta is the best. In terms of computational efficiency among the five robust methods, *t*Meta is still the most efficient while *t*RE-Meta and MIX-Meta demand the most time. Figure 6(d) shows the evolution of log-likelihood 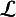 versus number of iterations when fitting *t*Meta. It can be seen from Figure 6(d) that *t*Meta requires 29 iterations to converge on this dataset.

The bottom row in Figure 7 shows the results of detecting outliers by the five methods. It can be seen that *t*Meta performs reliably as it successfully detects the two outliers: study 11, 8. In contrast, MIX-Meta and SKM-Meta fail to detect the most extreme study 11. *t*RE-Meta and SYM-Meta can identify study 11 but they fail to detect study 8.

## 5 Conclusion

For outlier accommodation and detection simultaneously, in this article we propose a novel robust meta-analysis model using student’s *t* distribution, namely *t*Meta. *t*Meta can be expressed as a hierarchical latent variable model while the marginal distribution of the effect size 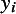 follows a tractable *t* distribution. To obtain the ML estimates of the parameters, we develop an ECME algorithm, which is computationally much more efficient than related methods as shown in our experiments. Empirical results on real datasets show that *t*Meta not only improves the robustness of nMeta as expected but also is compared favorably with closely related competitors in that it can provide the best performance for outlier accommodation and detection simultaneously, for both mild and gross outliers.

The experiment results show that SKM-Meta on some datasets yields better performance in outlier accommodation. For future work it would be interesting to extend *t*Meta using the skew-*t* distribution for further accommodating skewed data.

## Supporting information

Wang et al. supplementary material Wang et al. supplementary material

## Data Availability

The code to reproduce the results in our experiments is provided in the online supplementary materials and is also available on GitHub: the R code is available at https://github.com/wangyue4127/tmeta-R-code.git, and the MATLAB code can be found at https://github.com/wangyue4127/tmeta.git.

## Supplementary material

The supplementary material for this article can be found at https://doi.org/10.1017/rsm.2025.8.

## A Appendix

### A.1 The proposed ECME algorithm for *t*Meta

In this subsection, we develop an Expectation Conditional Maximization of Either (ECME) algorithm, a variant of the EM algorithm with faster monotone convergence. [15] Our ECME consists of an E-step followed by three conditional maximization (CM)-steps. In each CM step, a parameter in  is maximized while keeping the others fixed.

Let  be the missing data. From (3), the log-likelihood function of complete data  is given by

**E-step:** Compute the expected complete data log-likelihood function  with respect to the conditional distribution  ,

where, up to a constant

(A.1)

Here,  is given by (7). The required conditional expectation can be obtained by (9).

In our ECME, the first two CM-steps maximize  while the third CM-step maximize  . In detail,

**CM-step 1:** Given  , maximize  in (A.1) with respect to  yielding

(A.2)

**CM-step 2:** Given  , maximize  in (A.1) with respect to  under the same restriction  as in nMeta, [9] yielding

(A.3)

**CM-step 3:** Given  , maximize the observed data log-likelihood function  in (8) w.r.t.  . This is equivalent to finding the root of the following equation

(A.4)

where  , and  is the digamma function. Solving (A.4) only requires one-dimensional search, which can be performed, e.g., by the bisection method. [15]

For clarity, the complete ECME algorithm is summarized in Algorithm 1.

### A.2 Proof for Proposition 2

Proof. For ML estimate  , multiplying (9) by  , we obtain

(A.5)

On both sides of (A.3), multiply by  and then add  . On noting (7), when  , we have

(A.6)

and when  , we have

(A.7)

On both sides of (A.5), divide by  and take the sum over *i* from 1 to *N*, yielding

(A.8)

Substituting (A.6) and (A.7) into (A.8), respectively, we obtain, when  ,

(A.9)

and when  ,

(A.10)

When  , from (A.9) we have

and when  , from (A.10) we have

where  is given by (10). This completes the proof.
